# Phage-resistant *Pseudomonas aeruginosa* against a novel lytic phage JJ01 exhibits hypersensitivity to colistin and reduces biofilm production

**DOI:** 10.3389/fmicb.2022.1004733

**Published:** 2022-10-06

**Authors:** Wichanan Wannasrichan, Htut Htut Htoo, Rubsadej Suwansaeng, Joe Pogliano, Poochit Nonejuie, Vorrapon Chaikeeratisak

**Affiliations:** ^1^Department of Biochemistry, Faculty of Science, Chulalongkorn University, Bangkok, Thailand; ^2^Institute of Molecular Biosciences, Mahidol University, Nakhon Pathom, Thailand; ^3^Division of Biological Sciences, University of California, San Diego, La Jolla, CA, United States

**Keywords:** *P. aeruginosa*, bacteriophage, biofilm reduction, colistin susceptibility, phage resistance

## Abstract

*Pseudomonas aeruginosa*, a major cause of nosocomial infections, has been categorized by World Health Organization as a critical pathogen urgently in need of effective therapies. Bacteriophages or phages, which are viruses that specifically kill bacteria, have been considered as alternative agents for the treatment of bacterial infections. Here, we discovered a lytic phage targeting *P. aeruginosa*, designated as JJ01, which was classified as a member of the *Myoviridae* family due to the presence of an icosahedral capsid and a contractile tail under TEM. Phage JJ01 requires at least 10 min for 90% of its particles to be adsorbed to the host cells and has a latent period of 30 min inside the host cell for its replication. JJ01 has a relatively large burst size, which releases approximately 109 particles/cell at the end of its lytic life cycle. The phage can withstand a wide range of pH values (3–10) and temperatures (4–60°C). Genome analysis showed that JJ01 possesses a complete genome of 66,346 base pairs with 55.7% of GC content, phylogenetically belonging to the genus *Pbunavirus*. Genome annotation further revealed that the genome encodes 92 open reading frames (ORFs) with 38 functionally predictable genes, and it contains neither tRNA nor toxin genes, such as drug-resistant or lysogenic-associated genes. Phage JJ01 is highly effective in suppressing bacterial cell growth for 12 h and eradicating biofilms established by the bacteria. Even though JJ01-resistant bacteria have emerged, the ability of phage resistance comes with the expense of the bacterial fitness cost. Some resistant strains were found to produce less biofilm and grow slower than the wild-type strain. Among the resistant isolates, the resistant strain W10 which notably loses its physiological fitness becomes eight times more susceptible to colistin and has its cell membrane compromised, compared to the wild type. Altogether, our data revealed the potential of phage JJ01 as a candidate for phage therapy against *P. aeruginosa* and further supports that even though the use of phages would subsequently lead to the emergence of phage-resistant bacteria, an evolutionary trade-off would make them more sensitive to antibiotics.

## Introduction

*Pseudomonas aeruginosa* is a Gram-negative opportunistic pathogen responsible for nosocomial infections that represent a leading cause of Healthcare-Associated Infections (HAIs) (Magill et al., 2014; Weiner et al., 2016) and death in cystic fibrosis (CF) patients (Moradali et al., 2017). It also occupies the top position among Gram-negative bacteria as they are responsible for healthcare-associated pneumonia, the most common type of nosocomial infection in the United States (Magill et al., 2014). *P. aeruginosa* can naturally resist various classes of antibiotics due to its both intrinsic and extrinsic mechanisms for resistance, and thus, the treatment of *P. aeruginosa* infection tends to be less successful than other bacterial infections (Lister et al., 2009; Pang et al., 2019). Not only does it show intrinsic and acquired resistant mechanisms, *P. aeruginosa* also possesses an adaptive resistance in which the gene expression pattern is altered in response to stresses, growth stages, and environments (Breidenstein et al., 2011; Coleman et al., 2020). The ability of biofilm formation is one of the adaptive resistance mechanisms of *P. aeruginosa* whose biofilms serve as a virulence factor for its attachment to the surfaces and each other, assembling as a microenvironment. The biofilm also serves as a barrier that limits the diffusion of antibiotics, making it hard for them to reach the bacterial cells inside, further empowering its antibiotic resistance (Drenkard, 2003; Das et al., 2013). The drug-resistant *P. aeruginosa* has been announced by WHO as being “*first priority pathogens*” for global human health, for which new antibiotics or approaches are urgently needed (Duval et al., 2019). Unfortunately, the lack of discovery of novel drugs in comparison to the swift rate of the emergence of antibiotic resistance has made the fight against *P. aeruginosa* harder to handle (Gordillo Altamirano and Barr, 2019).

Also known as phage therapy, bacteriophages (phages), prokaryotic viruses that specifically kill bacteria, have been considered as an alternative way to combat drug-resistant bacteria (Kortright et al., 2019). The phages were claimed as the most abundant entities in the biosphere with a rough estimation of 10^31^ particles (Hatfull, 2015). Therefore, they can be easily found and used to fight against bacteria. Auto-dosing and specificity to the hosts are among the advantages of using phages in therapies. Phages have the ability to penetrate through biofilms, which are the drug-resistant mechanisms of *P. aeruginosa*, and kill persistent cells (Loc-Carrillo and Abedon, 2011). Phage therapies using *Pseudomonas* phages against *P. aeruginosa* infections have been proven to be successful in many cases worldwide, such as in the treatment of mouse models that were infected with the pathogen in multi-infection sites including lungs (Debarbieux et al., 2010; Morello et al., 2011; Chen et al., 2021; Yang et al., 2021), burn wounds (McVay et al., 2007), cornea (Fukuda et al., 2012), gut (Watanabe et al., 2007), and blood (Watanabe et al., 2007; Yang et al., 2021). Phage therapies for *P. aeruginosa* infection in humans have also been reported (Wright et al., 2009; Khawaldeh et al., 2011; Chan et al., 2018; Maddocks et al., 2019). For example, in England, a single dose of the six-phage cocktail was exploited to treat 24 patients with chronic otitis that was caused by antibiotic-resistant *P. aeruginosa*. After the therapy, the majority of the patients have significantly improved symptoms and also show a significant reduction in bacteria in patient samples (Wright et al., 2009). Due to recent breakthroughs in systematic phage therapy in clinical setup, many researchers have been focusing on the isolation of novel phages in order to create a strong foundation for phage therapy against this pathogen. As a result, many virulent *P. aeruginosa* phages have been discovered and characterized, giving rise to great potential for therapeutic purposes (Garbe et al., 2010, 2011; Pires et al., 2011; Ahiwale et al., 2012; Kim et al., 2012; Danis-Wlodarczyk et al., 2015; Yu et al., 2017; de Melo et al., 2019; Ong et al., 2020).

Albeit numerous successful cases of phage therapy against antibiotic-resistant bacterial infection, the development of phage-resistant *P. aeruginosa* after phage treatment is inevitable. A phage therapy study in endocarditis rats with a phage cocktail containing 12 phages revealed that the frequency of phage-resistant *P. aeruginosa* subpopulation occurred around 10^–4^ at 6 h and 10^–2^ at 24 h after phage treatment (Oechslin et al., 2016). *P. aeruginosa* is able to rapidly evolve to resist the phage infection through the modification of its outer membrane structure, such as glycosylation of its pilus and loss of *O*-antigen, where the phage first attaches (Johnson and Lory, 1987; Le et al., 2015; Yang et al., 2020). Hall et al. (2012) performed both single- and multi-phage therapy in wax moth larvae against *P. aeruginosa* infection, and they demonstrated that the single-phage use was not as effective as the multi-phage therapy to suppress bacterial growth. However, phage-resistant bacteria with impaired fitness were able to eventually emerge in both therapies (Hall et al., 2012). This raises concerns regarding the ability of *P. aeruginosa* which trades off its physiological fitness to rapidly evolve to resist phages (Hosseinidoust et al., 2013; Markwitz et al., 2021).

Since *P. aeruginosa* is highly capable of developing resistance to phages, it is very crucial to expand the variety of phages that effectively target this pathogen. This will allow scientists to have more choices to formulate efficient phage cocktails, which are highly preferred in the treatment of bacterial infections to suppress possible resistance (Chan et al., 2013). Here, we discovered a novel virulent *Pseudomonas* bacteriophage, named JJ01, which is classified as a member of *Pbunavirus* genus, *Myoviridae* family. This phage not only exhibits a strong lytic activity but also has the ability to eradicate biofilm formation of *P. aeruginosa*. Both conventional and molecular characterizations of phage JJ01 were conducted to explore the potential of the phage for therapeutic purposes, including adsorption assay, one-step growth experiment, burst size determination, viral particle stability, host range analysis, and whole-genome analysis. Moreover, JJ01-resistant *P. aeruginosa* strains were isolated and characterized for the physiological trade-off for phage resistance. Our finding revealed that this novel virulent *Pseudomonas* phage JJ01 could be a candidate for phage therapy in the future.

## Materials and methods

### Phage isolation, purification, and propagation

*Pseudomonas aeruginosa* strain PAO1 was used as the host for phage isolation. Before experiments, bacterial cells were grown overnight in Luria-Bertani (LB) broth (Tryptone; Himedia™, Cat. No. RM027, and Yeast extract; Himedia™, Cat. No. RM014) at 37°C with shaking at 250 rpm. Environmental samples were enriched by adding LB medium, 0.5 mM CaCl_2_, and *P. aeruginosa* culture, and incubated at 37°C for 48 h with shaking at 200 rpm. After the incubation, the mixture was centrifuged at 8,500 rpm and 4°C for 10 min and filtered through a 0.45 μm filter. A spot test was performed to indicate the existence of *P. aeruginosa* phage. For the phage purification, the filtrates were diluted in SM buffer and mixed with *P. aeruginosa* before preparing the double-layer agar (DLA) plates. After that, the single plaques were selected and kept in SM buffer. Plaques with the same morphology were selected and purified at least 3–5 times to ensure that identical phages were derived. To propagate high-titer bacteriophage, phage-containing SM buffer was diluted into a suitable dilution. Ten microliters of the phage suspension was mixed with the overnight culture of *P. aeruginosa* and 5 mL of 0.35% LB agar (top agar). Then, the mixtures were transferred onto LB plates (bottom agar). The plates that displayed web lysis of plaques were then selected and soaked with 5 mL of SM buffer for 5 h (Chaikeeratisak et al., 2017). After that, the phage lysate was collected and centrifuged at 8,500 rpm and 4°C for 10 min before filtration with a 0.45 μm filter. The phage lysate was kept at 4°C until use, and the phage titer was counted by spot test.

This work has been reviewed and approved by Chulalongkorn University-Institutional Biosafety Committee (CU-IBC) in accordance with the levels of risk in pathogens and animal toxins, listed in the Risk Group of Pathogen and Animal Toxin (2017) published by the Department of Medical Sciences (Ministry of Public Health), the Pathogen and Animal Toxin Act (2015) and Biosafety Guidelines for Modern Biotechnology BIOTEC (2016), with approval number: SC CU-IBC-028/2020 Ex1.

### Isolation and selection of phage-resistant *Pseudomonas aeruginosa*

Ten microliters of early-log phase PAO1 (OD_600_ ≈ 0.2) was mixed with 100 μL of 10^10^ PFU/mL of phage JJ01. The mixture was incubated for 5 min and diluted before spreading on LB plates (Le et al., 2015). After 24 h of incubation, the colonies were selected and purified at least three times. The phage resistance of the candidates was confirmed by drawing a straight line of each isolate on the LB agar plate and dropping 5 μL of phage lysate onto the bacterial stripe. After incubation, if a clear zone does not appear as observed in the wild-type PAO1, the bacterial isolates are considered to be phage-resistant. These isolates were then confirmed to be *P. aeruginosa* by MALDI Biotyper and were cryo-frozen for later experiments.

### Lysogeny test

Prophage induction by mitomycin C and prophage detection in bacterial host genomes were conducted to determine whether phage JJ01 was a temperate phage. In prophage induction by mitomycin C, mid-log phase cultures (OD_600_ ≈ 0.4) of JJ01-resistant isolates and wild-type PAO1 (internal control) were mixed with 10 μg/mL (final concentration) of mitomycin C. The mixtures were incubated at 37°C for 2 h before centrifugation at 12,000 × *g* for 5 min. The supernatants were filtered with 0.45 μm filters. The filtrates were serially diluted, followed by a spot test on the PAO1 lawn (Webb et al., 2003). The plates were incubated at 37°C for 24 h. Prophage detection in bacterial host genomes, colony PCR of genes gp44 and gp56 that are solely found in bacteriophages (particularly phage JJ01), was performed in phage-resistant *P. aeruginosa* isolates using specific primer sets. PCR products of gp44 and gp56 were then run on agarose gel electrophoresis and checked for the amplicon size. If a prophage integrates into the bacterial genome, its gene would be amplified and detected.

### Adsorption assay

To determine the time required for JJ01 attachment to *P. aeruginosa*, the bacterial culture with OD_600_ ≈ 0.4 (≈4 × 10^8^ CFU/mL) (Bucior et al., 2014) was infected with phage JJ01 at a multiplicity of infection (MOI) of 0.01 and incubated at 37°C. At each time point of incubation (0, 1, 2, 5, 7.5, 10, 15, 20, 25, and 30 min), the JJ01-PAO1 mixture was aliquoted and pelleted by centrifugation at 15,000 × *g* for 2 min. The supernatant was serially diluted in SM buffer, and a spot titer test was performed to determine the number of free phage particles in the supernatant. The experiment was performed in triplicates (Thammatinna et al., 2020).

### One-step growth curve

*Pseudomonas aeruginosa* PAO1 in exponential phase (OD_600_ ≈ 0.4) was infected with phage JJ01 at MOI of 0.01 for 10 min at 37°C. Then, the cells were centrifuged at 12,000 × *g* for 5 min. The pellet was resuspended in LB broth and incubated in a shaker at 250 rpm for 2 h. Every 10 min including time point at 0 min, the suspension was collected, serially diluted, and the phage titer was counted by plaque assay. The PFU/mL was calculated and plotted against time in minutes post-infection (mpi). The phage burst size was calculated from the plotted curve. Briefly, the average value of the highest phage titer after the burst was divided by the average value of baseline phage titer during the latent period. The result was described as particles per cell (Kropinski, 2018).

### pH and thermal stability

For thermal stability, 100 μL of phage JJ01 lysate was incubated at different temperatures: 4, 20, 30, 37, 40, 50, 60, 70, 80, and 100°C. For pH stability, 10 μL of phage JJ01 was mixed with 90 μL of different pH values of SM buffer ranging from pH 2 to 10. After 1 h of incubation, phage JJ01 from both experiments was serially diluted and enumerated by spot titer test. The experiments were performed in triplicates.

### Host range analysis

To determine the host of the phage JJ01, 5 μL of lysate was spotted on the lawn of *P. aeruginosa* strains derived from the Department of Medical Sciences (DMST), Ministry of Public Health, Thailand, and American Type Culture Collection (ATCC). Other hosts including *Vibrio cholerae*, *Escherichia coli*, *Burkholderia thailandensis*, and *Acinetobacter baumannii* were also used as indicated in Table 1. The efficiency of plating (EOP) was also performed in JJ01-susceptible bacteria by the spot test of different concentrations of phage JJ01 onto each tested bacterial lawn. The total number of plaques produced in each bacterial strain was then compared. The EOP was calculated (average PFU on target bacteria/average PFU on host bacteria) from triplicate along with the standard deviation. The average EOP value for a particular combination of phage and bacterium was classified according to the efficiency of killing as “High production” (the ratio was 0.5 or more), “Medium production” (the ratio was between 0.1 and 0.5), “Low production” (the ratio was between 0.001 and 0.1), and “inefficient” (the ratio was below 0.001) (Khan Mirzaei and Nilsson, 2015).

**TABLE 1:** Host range determination of phage JJ01.

Bacterial species	Strain	Source	Plaque formation	Efficiency of plating (EOP)
*Pseudomonas aeruginosa*	PAO1	Klockgether et al., 2010	+	1
	ATCC 9027	American type culture collection	–	
	ATCC 15442		+	0.89 (efficient)
	ATCC 27853		+	<.001 (inefficient)
*Pseudomonas chlororaphis*	200-B	Serwer et al., 2004	–	
*Pseudomonas stutzeri*	DMST 28410	DMST laboratory collection	–	
	DMST 12562		–	
*Pseudomonas mendocina*	ATCC 25411	American type culture collection	–	
*Pseudomonas fluorescens*	ATCC 13525		–	
*Pseudomonas putida*	ATCC 12633		–	
	ATCC 17522		–	
*Vibrio cholerae*	DMST 2873	DMST laboratory collection	–	
*Escherichia coli*	ATCC 25922	American type culture collection	–	
*Burkholderia thailandensis*	ATCC 700388		–	
*Acinetobacter baumannii*	ATCC 17978		–	
	ATCC 196096		–	

Different bacterial species and strains were used as the hosts to determine the host spectrum of the phage and efficiency of plating (EOP) using a spot test. DMST, Department of Medical Sciences, Ministry of Public Health, Thailand.

### Transmission electron microscopy

Phage JJ01 lysate (∼10^11^ PFU/mL) was precipitated overnight with 10% w/v polyethylene glycol and 1 M NaCl. The precipitated phage was pelleted at 8,500 rpm for 10 min and resuspended in SM buffer. The phage was negatively stained with 2% uranyl acetate on a carbon-coated grid, and the morphology of the phages was visualized by transmission electron microscopy (TEM) (HITACHI model HT7700). Then, the phages were classified based on their structure observed under TEM.

### Bacterial cell lysis assay

To determine the lytic activity of phage JJ01 against the host, *P. aeruginosa* PAO1 at the early exponential phase was infected with phage JJ01 in 96-well plates at different MOIs (0.01, 0.1, 1, 10, and 100) (Imam et al., 2019; Sharma et al., 2021). The mixtures were incubated at 37°C and were collected every 10 min for 12 h. During this, bacterial growth was monitored through the measurement of optical density (OD) using a Microplate reader (Synergy™ H1, Biotek) at 600 nm. The bacterial culture without phage infection was used as a control. The experiment was performed in triplicates.

### Biofilm eradication and formation assay

To conduct the biofilm eradication assay, 5 μL of overnight cultured *P. aeruginosa* PAO1 was seeded into each well of 96-well plates that contained 145 μL of LB broth. After incubation at 37°C for 48 h, planktonic cells were removed and the plate was washed two times with 300 μL of PBS buffer. Next, 200 μL of LB broth containing phage JJ01 (10^6^, 10^7^, and 10^8^ PFU/mL) was added to the wells, and the plate was further incubated at 37°C for 48 h. The phage suspension was then aspirated followed by washing with 300 μL of PBS buffer three times. To measure the biofilm amount remaining after eradication by the phage, 200 μL of absolute ethanol was added to the wells and the plate was then incubated for 15 min to fix the biofilm. After the ethanol was removed and dried, 200 μL of a 1% solution of crystal violet was added to the wells. The plate was incubated for 15 min at room temperature before rinsing with water three times in a water tub, followed by tapping on a paper towel to remove the excess water. After the plate was dried, 150 μL of 30% acetic acid solution was added to the wells, and the plate was further incubated for 1 h at room temperature to solubilize the crystal violet-staining biofilm. Finally, the absorbance of the biofilm content was measured at 580 nm using a Microplate reader (Synergy™ H1, Biotek). To measure biofilm formation of wild-type *P. aeruginosa* and JJ01-resistant isolates, 5 μL of bacterial overnight culture was added to 145 μL of LB broth. The cell suspension was cultured in the well plate at 37°C for 48 h, followed by the steps for biofilm measurement described above (Chaudhry et al., 2017; Bisht et al., 2021).

### Phage genomic DNA extraction

The DNA of phage JJ01 was extracted using phenol–chloroform–isoamyl alcohol method. Briefly, the phage lysate with at least 10^9^ PFU/mL was mixed with 10% w/v polyethylene glycol and 1M NaCl overnight to precipitate the phage particles. Phage precipitant was centrifuged at 8,500 rpm for 10 min (Sambrook et al., 1989). After that, it was re-suspended with SM buffer and treated with 10 U of DNase I and 0.1 mg/mL of RNase A to eliminate host DNA and RNA at 37°C. The DNase and RNase activities were inhibited by 20 mM EDTA, and the phage capsid was digested by 0.5 mg/mL of proteinase K and 0.5% sodium dodecyl sulfate (SDS). After the incubation at 55°C for 1 h, phenol–chloroform–isoamyl alcohol (25:24:1) (Sigma Aldrich, Switzerland) was added to the mixture at equal volumes. The mixture was gently mixed before centrifugation at 15,000 × *g* for 10 min, and the top aqueous layer was collected. The steps of phenol–chloroform–isoamyl alcohol and centrifugation were repeated before being treated with 1/10 volume of 3 M sodium acetate and 2 volumes of cold absolute ethanol. The genomic DNA was allowed to precipitate at –20°C for 3 h followed by centrifugation at 21,000 × *g* for 20 min. The DNA pellet was washed with 70% ethanol, centrifuged at 15,000 × *g* for 5 min, and the pellet was air-dried until the remaining ethanol evaporated. The phage DNA was dissolved in TE buffer. The DNA quality and quantity were analyzed using a NanoDrop 2000 spectrophotometer (Thermo Scientific) (Nale et al., 2016).

### Whole-genome sequencing, and genome and phylogeny analysis

Whole-genome sequencing (WGS) was carried out using the MiSeq (Illumina). FASTQ files derived from the sequencing process were assembled with SPAdes. The single contig of the phage genome was annotated with DNA master version 5.23.6 (Glimmer 3.02 and GeneMark HMM). The functional prediction was manually performed using viral genomes available in the NCBI database. PHASTER and NCBI conserved domains were additionally used for the annotation. The phage genome map was constructed and visualized using Artemis: DNAPlotter version 18.1.0. Genome phylogenetic tree was constructed by aligning phage JJ01 genome sequence against all *Pseudomonas* phage genomes designated as genus *Pbunavirus* approved by International Committee on Taxonomy of Viruses (ICTV), including NC_048676.1 (SL1), NC_011756.1 (SN), NC_048745.1 (vB_PaeM_SCUT-S1), NC_048663.1 (R26), NC_048662.1 (R12), NC_028939.1 (vB_Pae_PS44), NC_011810.1 (PB1), NC_048806.1 (PA8P1), NC_048626.1 (PA01), NC_011166.1 (LMA2), NC_041865.1 (phiKTN6), NC_007810.1 (F8), NC_048744.1 (EPa61), NC_042079.1 (vB_PaeM_E217), NC_042080.1 (vB_PaeM_E215), NC_041870.1 (vB_PaeM_CEB_DP1), NC_028971.1 (DL68), NC_028745.1 (DL60), NC_048675.1 (BrSP1), NC_026600.1 (vB_PaeM_C1-14_Ab28), NC_041902.1 (PA5), FM897211.1 (14-1), AB560486.1 (KPP12), JN254800.1 (NH-4), NC_048699.1 (vB_PaeM_LS1), NC_011165.1 (LBL3), and NC_017674.1 (JG024). The sequences were aligned with the iterative refinement method, and the tree was constructed using MAFFT version 7 with Neighbor-Joining algorithm (NJ) with Bootstrap 1,000x. The T4 phage genome was used as an outgroup to construct the phylogenetic tree. To elucidate the genetic relation between phage JJ01 and other phages in *Pbunavirus*, genome comparison was performed by Easyfig software version 2.1 (Imam et al., 2019). Pairwise intergenomic similarities between related phage genomes were performed using the Virus Intergenomic Distance Calculator (VIRIDIC^1^) (Moraru et al., 2020). The VIRIDIC algorithm relies on ICTV criteria to calculate intergenomic similarities among phages for classification.

### Minimum inhibitory concentration determination

Minimal inhibitory concentrations (MICs) were determined for the following antibiotics using the microdilution method: ciprofloxacin (CIP), piperacillin (CIP), tobramycin (TOB), colistin (COL), meropenem (MER), and gentamicin (GEN). The antibiotics were serially diluted in a 96-well plate. Overnight cultures of wild-type PAO1 and phage-resistant isolates were diluted 100-fold in LB and allowed to grow until they reached exponential growth (OD_600_ ≈ 0.2). The bacterial culture was further diluted in LB medium, in order to obtain 200,000 cells per well. The cells were dispensed to each well of a 96-well plate that contained different concentrations of antibiotics in LB. A positive growth control that contained bacteria without antibiotics and a negative media control of LB medium only were included for each antibiotic that was tested. The 96-well plate was then incubated at 30°C for 24 h. The MIC for each antibiotic was determined, in comparison with both the negative and positive controls, as the lowest concentration of antibiotic that could visibly inhibit the growth of bacteria. All MIC determinations were carried out at least in triplicates (Htoo et al., 2019).

### Fluorescence microscopy and image analysis

As previously described (Htoo et al., 2019), well-separated, single colonies of bacteria were inoculated in LB medium and grown overnight at 30°C on a roller at 50 rpm. The following day, the overnight culture was diluted 100-fold in fresh LB medium and further cultured until the early log phase of growth (OD_600_ of 0.2) was obtained. Colistin was added to the bacterial culture, at a concentration of 1.25 μg/mL, for both the wild-type PAO1 and W10 strains. Each colistin treatment was accompanied by an untreated control that consisted of the bacterial culture only. Both the control and the treated cultures were further incubated at 30°C on the roller at 50 rpm for 1 h. The cells were harvested by centrifugation at 8,000 x *g* for 30 s and concentrated by resuspending in 1:10 of the original volume. A few microliters of the concentrated culture was loaded onto a concave glass slide that was previously prepared with a 1.2% agarose pad on it, made with 10% LB, and containing fluorescent dyes: 1 μg/mL FM 4-64, 2 μg/mL DAPI, and 0.125 μM SYTOX Green. A cover slip was applied, and fluorescent microscopy was carried out on DeltaVision™ Ultra, with consistent imaging parameters throughout. Three independent experiments were performed for fluorescence microscopy. For image analysis, images from fluorescent microscopy were first pre-processed on the Fiji application (Schindelin et al., 2012), and then analyzed on CellProfiler software version 4.2.1 (Carpenter et al., 2006). From the parameters generated, a nucleoid outline was used to define the area from which SYTOX Green intensity is to be measured. The background intensity of each corresponding cell was then subtracted in order to obtain the intensity of SYTOX Green for each individual cell. Data for SYTOX Green intensity were obtained from the unadjusted, pre-deconvolved image from fluorescent microscopy. Data for the percentage of cells stained with SYTOX Green was collected from three independent experiments. Three to five images were randomly chosen from each condition (colistin-treated and untreated control) for every individual experiment. The percentage of cells stained with SYTOX Green was determined as the cells having SYTOX Green intensities more than twofold above (3x) the mean intensity of the untreated control for that strain and presented as mean ± standard deviation (STDEV).

### Statistical analysis

Statistical analysis was performed, and statistical significance was determined by student’s *t*-test and one-way ANOVA with Tukey’s HSD *post hoc* test. The analyses for each experiment are mentioned in the figure legends.

### Data availability

The nucleotide sequence of the *Pseudomonas* phage JJ01 genome was deposited in the GenBank database with the accession number: ON324181.1. All data needed to evaluate the conclusions in the article are present in the article and/or the Supplementary material.

## Results

### Morphological and biological properties of phage JJ01

**FIGURE 1:**
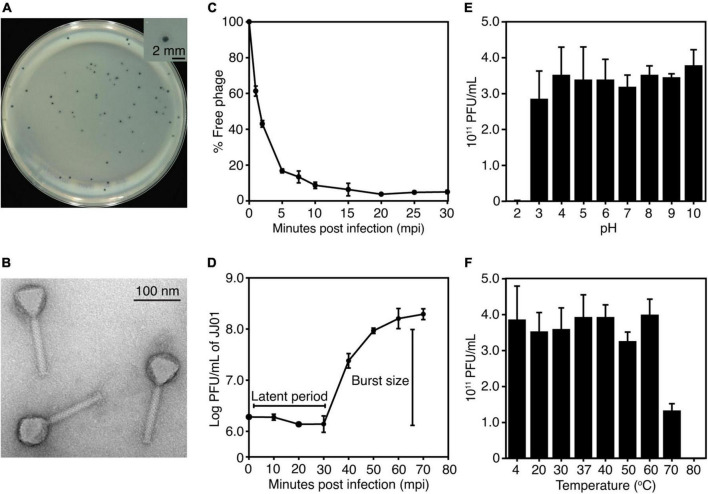
**(A)** Plaque morphology of phage JJ01. The right panel shows an individual plaque of JJ01. Scale bar equals 2 mm. **(B)** A transmission electron microscopy (TEM) image of the purified phage JJ01. Scale bar equals to 100 nm. **(C–F)** Biological properties of phage JJ01. **(C)** Adsorption assay, **(D)** One-step growth curve, **(E,F)** pH and thermal stability. **(C–F)** Experiments were performed in at least three replicates and data were represented as the mean ± standard deviation.

The newly isolated phage named “JJ01” was isolated from soil using *P. aeruginosa* PAO1 as a host. The individual plaque of phage JJ01 appears as either a clear or a turbid area at the center surrounded by a narrow opaque zone with a diameter of around 1.5 mm (Figure 1A and Supplementary Figure 1). As observed by TEM, phage JJ01 belongs to the *Myoviridae* family according to the structure that consists of an icosahedral capsid and a contractile tail, following the classification according to the International Committee on Taxonomy of Viruses (ICTV) guidelines. Phage JJ01 possesses an icosahedral head with an estimated height and width of 69.6 ± 3 nm and 65 ± 0.8 nm, respectively, and a tail that is approximately 118.9 ± 2.4 nm in length (Figure 1B; n = 5).

To better understand the biological properties of phage JJ01, we performed an adsorption assay, a one-step growth experiment, and a pH and thermal stability assay. The results of the adsorption assay demonstrated that phage JJ01 requires at least 10 min for around 90% of phage particles to get adsorbed onto the host cells (Figure 1C). One-step growth curve showed that phage JJ01 undergoes subcellular activities and propagates inside the host cells during the latent period, for 30 min, before releasing its progenies with a burst size of 109 particles per cell (Figure 1D). Furthermore, pH and thermal stability tests revealed that phage JJ01 withstands a wide range of pH from 3 to 10 and temperatures from 4°C to 60°C, where the numbers of viable phages are all comparable. Viable phages are completely inactivated at pH 2 and at 80°C; however, viable phages remain but decrease around 0.3 log at 70°C (Figures 1E,F). In addition, the host range of phage JJ01 was examined with 16 strains of various bacteria, including *Pseudomonas* spp. and other genera of bacteria. The result revealed that the phage is highly specific to *P. aeruginosa*, as it is capable of producing plaques only with *P. aeruginosa* PAO1, ATCC 15442, and ATCC 27853. No plaques were observed in other *Pseudomonas* strains and bacteria of different genera (Table 1).

### Genome features and annotation of phage JJ01

**FIGURE 2:**
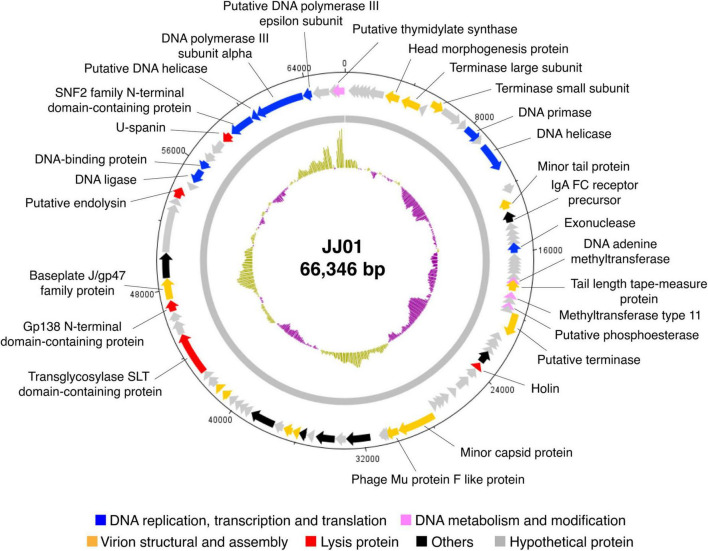
Circular genomic map of phage JJ01. The genome size is 66,346 base pairs, composed of 92 ORFs which are functionally annotated and are divided into different groups as indicated by colors as follows: blue, DNA replication and transcription, and translation; pink, DNA metabolism and modification; orange, virion structural and assembly; red, lysis protein; black, others; and gray, hypothetical proteins. An inner plot presents the GC content across the genome (yellow above average and purple below average). The direction of arrows indicates gene arrangement in the JJ01 genome. The map was generated using Artemis: DNAPlotter version 18.1.0.

Whole-genome sequencing by Illumina platform resulted in 1,287,392 reads of FastQ sequence after host DNA subtraction. After assembly by SPAdes with coverage over 2000x, a complete genome of phage JJ01 was obtained. Phage JJ01 has a double-stranded circularly permuted genome. Its genome is 66,346 base pairs long with a GC content of 55.7% and encodes 92 open reading frames (ORFs), as annotated by DNA master software (Figure 2 and Supplementary Table 2). For functional annotation, 38 ORFs with significant hits (*E*-values less than 10^–5^ according to Blastx search) were designated as functionally predicted genes (Table 2), while the rest (54 ORFs) were identified as hypothetical proteins. The functionally predicted ORFs were found to be composed of 9 genes related to DNA replication, transcription, and translation; 4 genes related to DNA metabolism and modification; 13 genes related to virion structure and assembly; 5 genes related to host cell lysis proteins; and 7 genes for other phage-related proteins. tRNA-encoding genes were not found in the genome (Figure 2 and Table 2). Phage JJ01 encodes a set of necessary enzymes involved in DNA replication (e.g., DNA primase; ORF13, DNA helicase; ORF15, DNA ligase; ORF81, and DNA polymerase III alpha and epsilon subunits; ORF88 and ORF89), DNA metabolism and modification (e.g., DNA adenine methyltransferase, ORF30, and thymidylate synthase, ORF92). Proteins involved in the DNA packaging machinery, including terminase large (ORF8) and small (ORF10) subunits, and the cell lysis module, including holin (ORF42), transglycosylase SLT domain-containing protein (ORF71), endolysin (ORF79), and *U*-spanin (ORF85), were also identified in the genome. In addition, toxin, drug-resistant, or lysogenic-associated genes were not found in the JJ01 genome. Since our genome annotation suggested that phage JJ01 is potentially a lytic phage due to the absence of lysogenic-associated genes in the genome, we therefore validated our annotation by the lysogeny test. We first isolated 10 JJ01-resistant *P. aeruginosa* strains and then confirmed the phage resistance and bacterial species by MALDI Biotyper (Supplementary Figure 2). After the activation of lysogens by mitomycin C, no clear zone was found around the spot of all JJ01-resistant *P. aeruginosa* isolates, indicating that no phage is released from the bacterial cells after stimulation (Supplementary Figure 3). To further confirm that phage JJ01 strictly enters the lytic cycle and is not capable of integrating its genome into the host cells, we screened for JJ01 prophage in the genomes of the resistant isolates using primer sets specific to ORF44 and ORF56 (Supplementary Figure 3). The result revealed that none of the resistant strains contain an integrated genome of phage JJ01, suggesting that phage JJ01 is indeed a lytic bacteriophage.

**TABLE 2:** List of functionally annotated proteins from ORFs in the genome of phage JJ01.

ORF	Predicted function	Direction	Start	Stop	Size (n)	Sequence similarity	Accession no.	Database
ORF7	Head morphogenesis protein	–	2533	3423	891	PHAGE_Pseudo_vB_PaeM_E217 _NC_042079: head morphogenesis protein	PP_00007	PHASTER
ORF8	Terminase large subunit	–	3584	4771	1188	PHAGE_Pseudo_vB_PaeM_SCUT _S1_NC_048745: terminase large subunit	PP_00008	PHASTER
ORF10	Terminase small subunit	+	5349	6134	786	PHAGE_Pseudo_vB_PaeM_E217 _NC_042079: terminase small subunit	PP_00009	PHASTER
ORF13	DNA primase	+	7853	8929	1077	RepB family DNA primase [*Staphylococcus aureus*]	WP_015994935.1	NCBI
ORF15	DNA helicase	+	9268	11007	1740	DNA helicase [*Pseudomonas* phage Epa20]	QIQ65309.1	NCBI
ORF17	Minor tail protein	–	12714	13325	612	PHAGE_Pseudo_vB_PaeM_LS1 _NC_048699: minor tail protein	PP_00016	PHASTER
ORF18	IgA FC receptor precursor	–	13514	14200	687	IgA FC receptor precursor [*Pseudomonas* phage debbie]	QIQ66578.1	NCBI
ORF23	Exonuclease	-	15478	16122	645	PHAGE_Pseudo_vB_PaeM_LS1 _NC_048699: exonuclease	PP_00022	PHASTER
ORF30	DNA adenine methyltransferase	–	17616	17804	189	PHAGE_Pseudo_vB_PaeM_LS1 _NC_048699: DNA adenine methyltransferase	PP_00030	PHASTER
ORF31	Tail length tape-measure protein	–	17807	18493	687	Tail length tape-measure protein [*Pseudomonas* phage debbie]	QIQ66591.1	NCBI
ORF32	Methyltransferase type 11	–	18546	18848	303	PHAGE_Pseudo_vB_PaeM_SCUT _S1_NC_048745: methyltransferase type 11	PP_00032	PHASTER
ORF34	Putative phosphoesterase	–	19203	19367	165	PHAGE_Pseudo_vB_PaeM_LS1 _NC_048699: putative phosphoesterase	PP_00035	PHASTER
ORF36	Putative terminase	+	19772	21154	1383	Putative terminase [*Pseudomonas* phage KPP12]	YP_007238156.1	NCBI
ORF41	DUF2786 protein	–	22569	23348	780	[pfam10979] cl12553 (PSSM Id: 402523) Protein of unknown function	cl12553	NCBI Conserved Domain Search
ORF42	Holin	–	23435	23872	438	PHAGE_Pseudo_Epa13 _NC_050147: holin	PP_00043	PHASTER
ORF50	Minor capsid protein	+	27664	29961	2298	Minor capsid protein [*Pseudomonas* phage vB_PaeM_USP_1]	YP_009914222.1	NCBI
ORF51	Phage Mu protein F like protein	+	29961	30797	837	[Phage_Mu_F super family] cl10072 (PSSM Id: 415838) Phage Mu protein F like protein	cl10072	NCBI Conserved Domain Search
ORF54	DUF2213 protein	+	31672	33105	1434	[DUF2213 super family] cl19842 (PSSM Id: 418671) Uncharacterized protein conserved in bacteria	cl19842	NCBI Conserved Domain Search
ORF56	DUF2184 protein (major capsid)	+	33754	34902	1149	[DUF2184 super family] cl21556 (PSSM Id: 419730) Uncharacterized protein conserved in bacteria	cl21556	NCBI Conserved Domain Search
ORF58	DUF4054 domain-containing protein	+	35456	35923	468	DUF4054 domain-containing protein [*Pseudomonas aeruginosa*]	WP_016066139.1	NCBI
ORF59	Putative structural protein	+	35920	36318	399	Putative structural protein [*Staphylococcus aureus*]	WP_016066140.1	NCBI
ORF60	Structural protein	+	36326	36877	552	Structural protein [*Pseudomonas* phage phiKTN6]	YP_009593265.1	NCBI
ORF62	DUF3383 domain-containing protein	+	37471	38985	1515	DUF3383 domain-containing protein [*Staphylococcus aureus*]	WP_174840936.1	NCBI
ORF67	Putative structural protein	+	40609	41112	504	Putative structural protein [*Pseudomonas* phage KPP12]	YP_007238188.1	NCBI
ORF68	Structural protein	+	41247	41651	405	Structural protein [*Pseudomonas* phage goonie]	QJB23037.1	NCBI
ORF71	Transglycosylase SLT domain-containing protein	+	42695	45271	2577	Transglycosylase SLT domain cl00222 (PSSM Id: 396169)	cl00222	NCBI Conserved Domain Search
ORF74	Gp138 N-terminal domain-containing protein	+	46723	47388	666	[Gp138_N super family] cl39697 (PSSM Id: 423479) Phage protein Gp138 N-terminal domain	cl39697	NCBI Conserved Domain Search
ORF75	Baseplate J/gp47 family protein	+	47446	48699	1254	Baseplate J/gp47 family protein [*Pseudomonas aeruginosa*]	MBI7739469.1	NCBI
ORF76	DUF2612 protein	+	48696	50210	1515	[DUF2612 super family] cl12607 (PSSM Id: 416601) Protein of unknown function	cl12607	NCBI Conserved Domain Search
ORF79	Putative endolysin	+	53539	54201	663	Putative endolysin [*Pseudomonas* phage KPP12]	YP_007238200.1	NCBI
ORF81	DNA ligase	–	54757	55668	912	DNA ligase [*Pseudomonas* phage PHW2]	QKW95294.1	NCBI
ORF82	DNA-binding protein	–	55723	56277	555	DNA-binding protein [*Pseudomonas* phage debbie]	QIQ66549.1	NCBI
ORF85	U-spanin	–	57924	58544	621	PHAGE_Pseudo_vB_PaeM_E215 _NC_042080: u-spanin	PP_00086	PHASTER
ORF86	SNF2 family N-terminal domain-containing protein	–	58639	60198	1560	[SNF2_N Superfamily] cl37620 (PSSM Id: 422040) SNF2 family N-terminal domain	cl37620	NCBI Conserved Domain Search
ORF87	Putative DNA helicase	–	60195	60605	411	Putative DNA helicase [*Pseudomonas* phage NH-4]	YP_007002602.1	NCBI
ORF88	DNA polymerase III subunit alpha	–	60598	63708	3111	DNA polymerase III subunit alpha [*Staphylococcus aureus*]	WP_174840946.1	NCBI
ORF89	Putative DNA polymerase III epsilon subunit	–	63705	64259	555	Putative DNA polymerase III epsilon subunit [*Pseudomonas* phage NH-4]	YP_007002604.1	NCBI
ORF92	Putative thymidylate synthase	–	65550	66338	789	Putative thymidylate synthase [*Pseudomonas* phage PaGU11]	YP_009913899.1	NCBI

The predicted functions of ORFs were determined by their significant hit (*E*-value < 10^–5^) against the genome in NCBI and PHASTER.

### Phylogenetic tree and genome comparison of JJ01 against *Pbunavirus*

**FIGURE 3:**
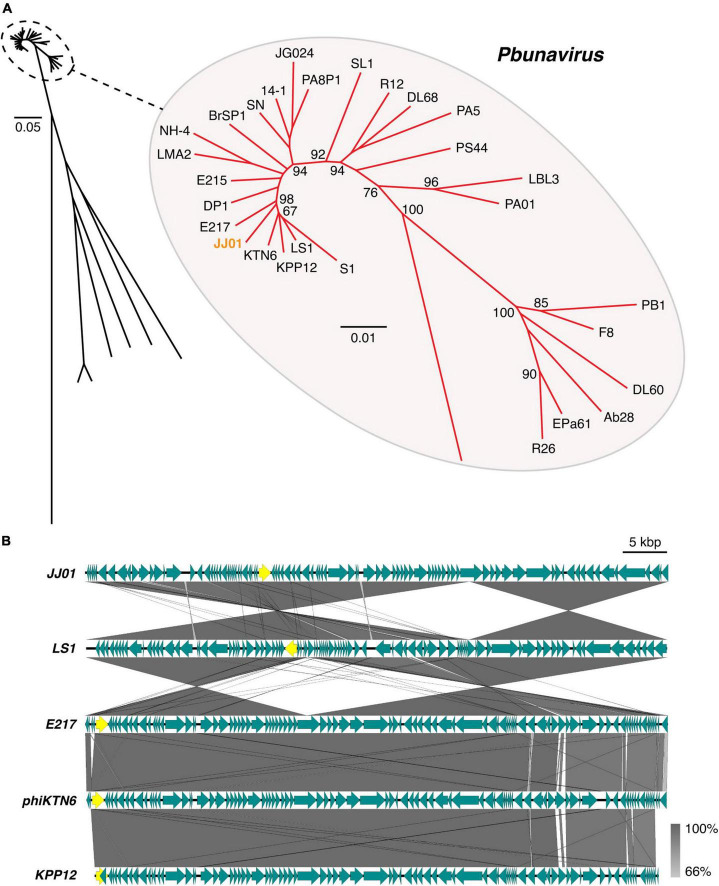
**(A)** Genome phylogenetic tree between species in *Pbunavirus* according to ICTV approval constructed using MAFFT version 7 with 1,000x Bootstrap. Numbers at branches represent Bootstrap values (%). Scale bars represent the branch length of taxa. **(B)** Comparative genomic analysis shows the gene organization of phage JJ01 compared to phages LS1, E217, phiKTN6, and KPP12 whose genomes are closely related to phage JJ01. Yellow labeled arrows represent the location and direction of the large subunit terminase gene.

According to Blastn search, the JJ01 genome sequence is highly similar to those of the members of the genus *Pbunavirus* (Order *Caudovirales*; Family *Myoviridae*). Genome to genome phylogenetic tree of phage JJ01 and other phages in the *Pbunavirus* genus was therefore constructed. The results showed that JJ01 has a very close relationship with phages LS1, E217, phiKTN6, and KPP12 (Figure 3A). VIRIDIC program was used to calculate pairwise intergenomic similarities among the phages, and it revealed that the genome similarity of phage JJ01 against KPP12, phiKTN6, E217, and LS1 is 93.89, 95.46, 96.04, and 96.44%, respectively (Supplementary Table 1). Even though all were clustered into the same genus, phage JJ01 was classified into a different species from KPP12 and phiKTN6. However, JJ01, E217, and LS1 were clustered into the same species due to the very high genome similarity (more than 96%) (Supplementary Table 1). To further elucidate whether their genomes are similarly organized, complete genomes of phages JJ01, E217, and LS1 were compared. The result revealed that, although JJ01, E217, and LS1 are likely the same species, based on genome similarity, their genomes are differently organized (Figure 3B). Specifically, the gene encoding for a terminase large subunit (Figure 3B, yellow arrow) of JJ01 is located at a different locus, compared to that of the other phages. Moreover, the large subunit of the terminase gene of LS1 is found to be located on the complementary strand of DNA, which is opposite to the direction of the JJ01 terminase gene. Altogether, these results suggested that phage JJ01 is a novel *Pseudomonas* phage that belongs to the genus *Pbunavirus*, whose genome sequence is highly similar to phages E217 and LS1.

### Phage JJ01 efficiently suppresses bacterial growth and eliminates biofilm production

**FIGURE 4:**
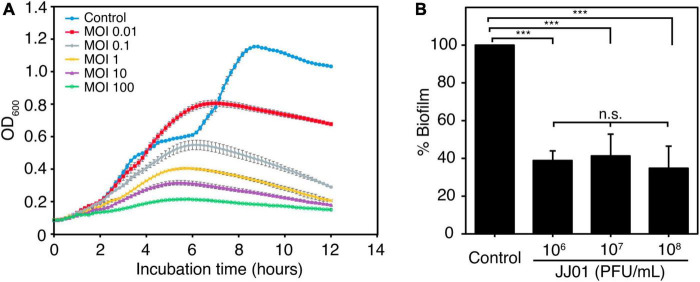
**(A)** Killing assay shows the ability of phage JJ01 to lyse *P. aeruginosa* cells at different multiplicity of infections (MOIs) at 0.01, 0.1, 1, 10, and 100 in LB medium at 37*^o^*C for 12 h. Each experiment was performed in triplicate. Data and error bars were represented as the mean ± standard deviation. **(B)** Biofilm eradication assay. Crystal violet staining reveals the grown biofilm level of *P. aeruginosa* PAO1 after incubation with phage JJ01 at 10^6^, 10^7^, and 10^8^ PFU/mL for 48 h compared with the control (absence of phage JJ01). Asterisks represent significant differences in data (*p* ≤ 0.001) according to one-way ANOVA followed by Tukey’s HSD *post hoc* tests. n.s. represents statistical non-significance of data.

To determine the killing activity of phage JJ01 against *P. aeruginosa* PAO1, we measured the bacterial growth during phage infection for 12 h at different MOIs (Figure 4A). The results demonstrated that MOI at 100 appears to be the best ratio to inhibit and efficiently suppress the growth of *P. aeruginosa* throughout the period of 12 h. As expected, the lower the MOIs, the lower the lytic activity. The graph showed descending lytic activities of the phage at MOIs from 10 to 0.01, with a pattern of bacterial growth quite similar to an MOI of 100. OD values of the cultures at MOI from 10 to 0.01 only increase at the beginning of incubation until 6 h, followed by a gradual decline until the end of the experiment (Figure 4A). In addition, during the experiment of 12 h, phage-resistant *P. aeruginosa* does not seem to emerge. To further investigate whether phage JJ01 plays any role in eliminating biofilm production of the bacteria, we first allowed *P. aeruginosa* to produce biofilm followed by the phage addition at various titers (10^6^-10^8^ PFU/mL) to test the ability of phage to eradicate the biofilm. The result revealed that phage JJ01 is able to eliminate the established biofilms with an almost 3-fold reduction when compared to the control (Figure 4B). Our findings that *Pseudomonas* phage JJ01 is highly efficient not only in killing the bacteria but also in eradicating the biofilm formation of the bacteria. These demonstrate the potential use of phage JJ01 in the clinical setting.

### Characterization of physiological properties of JJ01-resistant *Pseudomonas aeruginosa*

**FIGURE 5:**
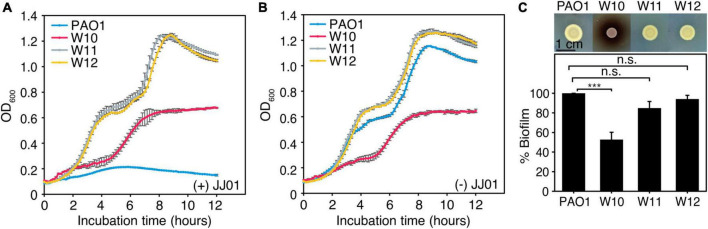
**(A,B)** Growth curve of wild-type PAO1 and representatives of phage-resistant isolates (W10, W11, and W12) **(A)** in the presence of phage JJ01 at MOI 100 and **(B)** in the absence of phage JJ01. **(C)** Colony morphology of JJ01-resistant *P. aeruginosa* isolates compared to wild-type PAO1 (upper panel). Scale bar equals 1 cm. The biofilm production assay shows the amount of biofilm production in JJ01-resistant *P. aeruginosa* isolates (W10, W11, and W12) compared to wild-type PAO1 (lower panel). Asterisks represent significant differences in data (*p-value* ≤ 0.001) according to one-way ANOVA followed by Tukey’s HSD *post hoc* tests. n.s. represents statistical non-significance of data.

Even though phage resistance was not detected during infection at 12 h (Figure 4A), the emergence of JJ01-resistant *P. aeruginosa* was observed after incubation with phage JJ01 overnight. Previous studies showed that, due to an evolutionary trade-off, bacterial resistance to certain stressors required drastic physiological changes that commonly led to some degree of vulnerabilities, such as reduced bacterial overall fitness and susceptibility toward antibiotics (Le et al., 2015; Pires et al., 2017; Markwitz et al., 2021). Thus, we first explored if resistance to the phage affects any aspects of bacterial growth and physiology. Among the 10 JJ01-resistant strains (Supplementary Figures 2, 3), we selected JJ01-resistant strains W10, W11, and W12 as representatives of the study. We sequenced the whole genomes of these isolates, and the genomic data showed no sequences of JJ01 prophage in the genomes, assuring that W10, W11, and W12 are not lysogens and truly phage-resistant *P. aeruginosa* (data not shown). By measuring a bacterial growth curve in the presence of phage JJ01 at MOI of 100, the growth of the wild-type isolate is completely suppressed by phage JJ01 (Figures 5A,B; blue line). However, the growth of W10, W11, and W12 is not affected by the phage, indicating that the phage is not capable of killing the bacteria (Figures 5A,B; red, gray, and yellow lines). The trade-off of resistance to the phage seems to affect the growth and physiological properties of the isolates. Among the three representatives of resistant isolates, even though W11 and W12 appear to grow faster than the wild type, W10 grows much slower (Figure 5B; red line). The growth of W10 was also confirmed by smaller colony size when compared to the others (Figure 5C; upper panel). The defect in the bacterial growth was also observed in the other JJ01-resistant isolates, which accounted for approximately 85% of the isolates (six out of the additional seven tested isolates) (Supplementary Figure 4A). Moreover, some resistant isolates are impaired in their ability to produce biofilm, as W10, W132, W138, W142, and W143 produce up to 3-fold less biofilm than the wild-type PAO1 (Figure 5C, lower panel and Supplementary Figure 4B), and none is capable of producing higher amount of biofilm than the wild type statistically. Altogether, these results suggested that, to gain resistance toward the phage JJ01, some bacteria undergo a physiological trade-off that would result in the defect in bacterial growth and biofilm production.

### Resistance to phage JJ01 affects antibiotic susceptibility and increases cell permeability by colistin

**FIGURE 6:**
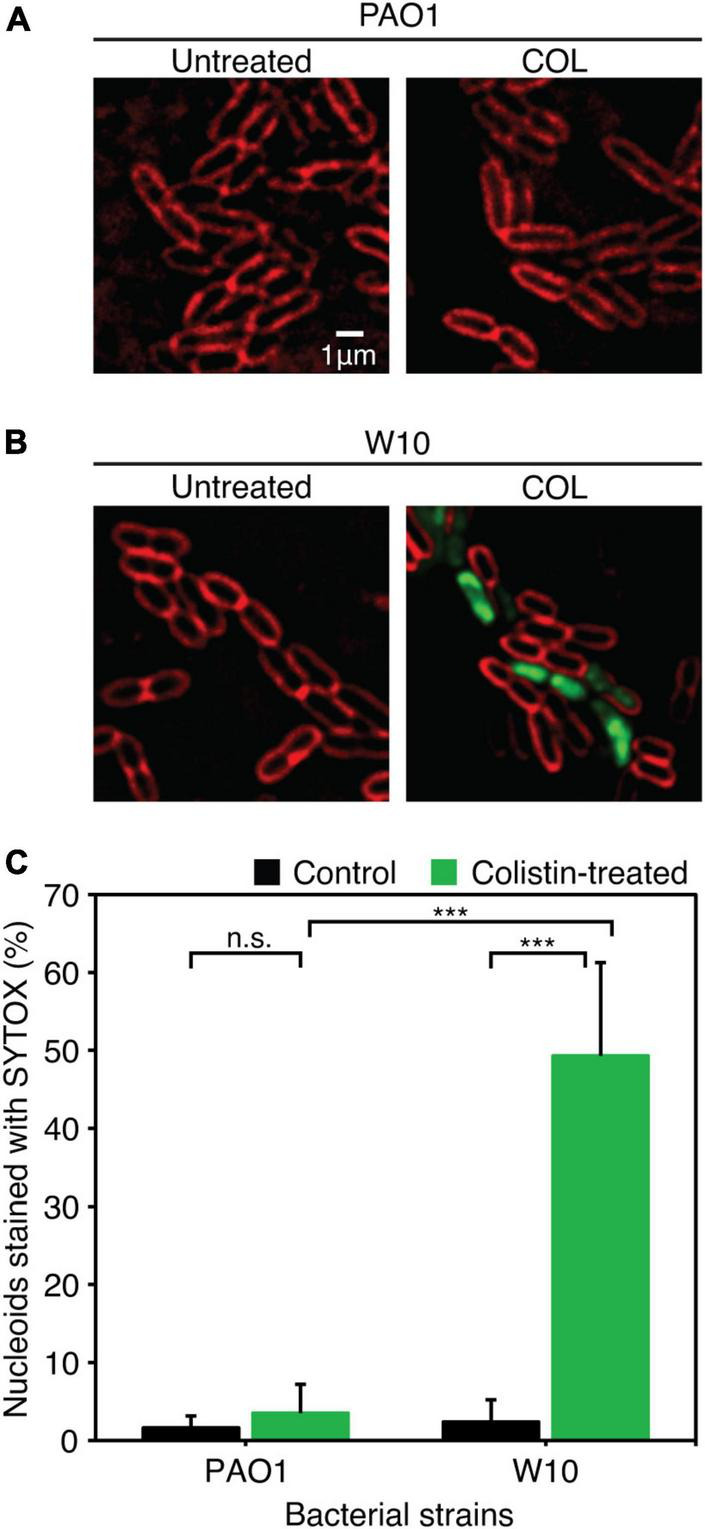
**(A,B)** Fluorescence images of wild-type PAO1 **(A)** and phage-resistant isolates (W10) **(B)** upon treatment with COL. The cell membrane was stained with FM4-64 (red) and nucleoid of cells with impaired membrane integrity was stained with impermeable SYTOX (Green). **(C)** Percentage of nucleoids strained with SYTOX Green after treatment with COL in wild-type PAO1 and phage-resistant isolation (W10) compared to untreated cells. SYTOX Green was used to stain DNA as an indicator for the cells with permeabilized membranes. Asterisks represent significant differences in data at *p* ≤ *0.001* according to the student’s *t*-test and n.s. represents statistical non-significance of data. The test was performed in triplicate. Scale bar equals 1 μm.

Because biofilm serves as a barrier that limits the diffusion of antibiotics to access the bacterial cells (Pang et al., 2019), it was tempting to explore if the phage-resistant isolates that have lost their capability to produce biofilms become more sensitive to antibiotics. We then performed a minimum inhibitory concentration (MIC) assay of JJ01-resistant isolates and wild-type PAO1 against six antibiotics, which are commonly used to treat *P. aeruginosa* infections (Table 3 and Figure 6). The result showed that JJ01-resistant strains display various drug susceptibility profiles in the range of 2x increase (PIP, TOB, and MER) and 2x decrease (CIP, COL, MER, and GEN) in MIC, compared to those of the wild-type PAO1. Nevertheless, by the definition of Clinical and Laboratory Standard Institute (CLSI) standard, none of the JJ01-resistant isolates is defined as resistant to antibiotics according to their MIC level. Interestingly, 6 out of 10 resistant strains become 4–8 times more susceptible to COL as indicated by their lower MIC levels (Table 3). Among these strains, W10 and W132, which were observed to grow slower and produce brownish pigments, were noticeably more sensitive to COL with an 8-fold decrease in MIC, compared to wild-type PAO1 (Figure 5C, Supplementary Figures 2A, 4A, and Table 3). The hypersensitivity of W10 to COL was further confirmed by single-cell death analysis via impermeable SYTOX Green staining. In principle, when the membrane is compromised by COL, SYTOX Green can enter the cells and stain the nucleoid, thus emitting green fluorescence signals (Htoo et al., 2022). In other words, the more the cell membranes are damaged, the higher the number of cells that emit green fluorescence can be observed. This sensitivity of W10 toward COL, as tested in MIC assay, is in agreement with our microscopy data. In the untreated wild-type and W10 strains (Figures 6A,B; left panels), SYTOX Green did not well stain the bacterial nucleoids of both PAO1 and W10, as indicated by the very low percentage of stained nucleoids at 1.605 ± 1.522 and 2.369 ± 2.856, respectively (Figure 6C), indicating the uncompromised bacterial cell barrier. During the treatment of COL in the wild-type PAO1, the percentage of positive staining slightly increased, when compared to the untreated condition (Figures 6A,C). However, treatment of W10 with COL resulted in a substantial increase (above 14 times higher) in the percentage of stained nucleoids than in the treated wild-type PAO1 (Figures 6A–C), suggesting that the cell membrane of the majority of the W10 population was compromised and thus is more fragile than the wild-type cells when exposed to the same concentration of COL. Altogether, the results demonstrated that the evolution of phage resistance of W10 against phage JJ01 affects bacterial physiological properties, some of which, in turn, lead to antibiotic hypersensitivity of the bacteria.

**TABLE 3:** Minimum inhibitory concentration (MIC) determination of six main antibiotics used for the treatment of *P. aeruginosa* infection, ciprofloxacin (CIP), piperacillin (PIP), tobramycin (TOB), colistin (COL), meropenem (MER), and gentamicin (GEN), against wild-type PAO1 and 10 strains of phage-resistant isolates.

Strains	CLSI susceptibility (MIC, μg/mL)					
	CIP	PIP	TOB	COL	MER	GEN
PAO1	S (0.08)	S (2.50)	S (1.25)	S (1.25)	S (0.62)	S (2.50)
W10	S (0.04)	S (5.00)	S (1.25)	S (0.16)	S (1.25)	S (1.25)
W11	S (0.04)	S (2.50)	S (1.25)	S (1.25)	S (1.25)	S (1.25)
W12	S (0.04)	S (2.50)	S (1.25)	S (1.25)	S (1.25)	S (1.25)
W132	S (0.08)	S (5.00)	S (2.50)	S (0.16)	S (0.62)	S (1.25)
W138	S (0.04)	S (5.00)	S (2.50)	S (0.31)	S (0.31)	S (2.50)
W139	S (0.04)	S (5.00)	S (2.50)	S (0.31)	S (0.62)	S (1.25)
W140	S (0.04)	S (5.00)	S (2.50)	S (0.31)	S (0.62)	S (1.25)
W141	S (0.04)	S (5.00)	S (2.50)	S (0.31)	S (0.31)	S (1.25)
W142	S (0.04)	S (5.00)	S (2.50)	S (0.62)	S (0.31)	S (2.50)
W143	S (0.04)	S (2.50)	S (1.25)	S (1.25)	S (0.62)	S (2.50)

The test was performed in triplicate and categorized based on the drug susceptibility level; S, sensitive; I, intermediate; R, resistant, according to CLSI standards (CLSI, 2020). Gray (wild-type MIC level), yellow (2X increase), light green (2X decrease), medium green (4X decrease), and dark green (8X decrease).

## Discussion

The emergence of antibiotic-resistant *P. aeruginosa* together with the shortage of novel antibiotic discoveries urges scientists around the world to find other alternative ways to deal with this worsening situation. Using bacteriophages for therapeutic purposes is one of the alternatives that have been proven to be a promising tool to cope with drug-resistant bacteria (Schooley et al., 2017; Dedrick et al., 2019). Here, we successfully isolated a novel virulent *P. aeruginosa* phage, designated as JJ01, from soil and showed its characteristic feature, which can be classified as a myovirus in the *Myoviridae* family. The myoviruses constitute about 25% of all tailed phages in the *Caudovirales* order (Ackermann, 2003), and are frequently found in Gamma-proteobacteria, the phylum of *Pseudomonas* genus. According to VIRIDIC analysis among phages in the *Pbunavirus* genus, JJ01 is very closely related to the phage E217 (96.04%) and with the highest similarity toward LS1 (96.44%). As the ICTV species demarcation criteria that classify the genome sequences whose sequence similarity is above 95% as organisms in the same species (Adriaenssens and Brister, 2017; Moraru et al., 2020), this raises some concerns on whether or not the phage JJ01 is a novel phage. Phylogenetic tree analysis revealed that JJ01 and E217 are classified as different species because they are in separate branches of the tree with a strong-supporting bootstrap value of over 95% (Soltis and Soltis, 2003). Even though the tree weakly supports the claim of JJ01 and LS1 as different species since the bootstrap number on their nodes is lower than 95 but higher than 50 (Soltis and Soltis, 2003), genome comparison between these phages indeed assured that they are different due to the distinct genome organizations. Our finding is not the only case. It is possible that different *Pseudomonas* phages isolated at different periods and from distant geographical areas contain high genome sequence similarities. For example, distinct giant phages that were classified in different families (siphophage SD1-M and nucleus-forming myophage phiKZ) shared a very high genome identity up to 99% with only two DNA segments different between their genomes (Kwan et al., 2006; Krylov et al., 2007; Chaikeeratisak et al., 2021). Specifically, in the *Pbunavirus* genus, PB1-like phages, which are the very common virulent *P. aeruginosa* phages, shared a remarkable genome similarity to one another (over 95%). Despite the very high similarity of their genomes, they are organized in different patterns and contain unequal gene clusters (Ceyssens and Lavigne, 2010). Therefore, our finding further supports this statement and revealed that the genome organization of phage JJ01, as one of the *Pbunavirus* members, has its gene orientation and genome organized differently from the nearest-related phage, LS1.

In the attempt to employ phages for therapeutic purposes for *P. aeruginosa* infection, a variety of *Pseudomonas* phages and phage cocktails have been isolated and investigated for their roles in the treatment of the infections (Wright et al., 2009; Khawaldeh et al., 2011; Chan et al., 2018; Maddocks et al., 2019). Many *Pseudomonas* phages in *Pbunavirus* genus have been proven to be successful in phage therapy. For example, phage KPP12, whose genome is highly similar to JJ01 (93.89%), displayed a broad host range over the clinical isolates of *P. aeruginosa*, and the phage use by single-dose administration was proven to improve the symptom outcome of ophthalmic infections in mice (Fukuda et al., 2012). Phages E215 and E217 which are genetically close to JJ01 were used as ingredients of a six-phage cocktail to treat acute respiratory infection in mice and cure bacteremia in wax moth larvae infected with *P. aeruginosa*. The phage cocktail was able to rescue and prolong the survival of the treated animals, suggesting the high efficiency of the cocktail (Forti et al., 2018). Our novel *P. aeruginosa* phage JJ01 does also display suitable characteristics and would be considered as a decent candidate for therapy. The phage JJ01 is a lytic bacteriophage. The phage is highly specific to *P. aeruginosa* and is capable of killing 75% of the tested strains of *P. aeruginosa*. With the fast adsorption to the bacterial host cells, it spends relatively short periods of replication and produces many phage progenies. Genome annotation further demonstrated that lysogenic-associated genes, as well as other unwanted genes such as toxin, virulence, or antibiotic-resistant genes, are not present in the JJ01 genome. The lysogeny test experimentally confirmed that JJ01 is not capable of lysogenizing the host and strictly enters the lytic cycle. Together with the stability of the phage over broad ranges of pH and temperatures, we have assured that phage JJ01 is potentially a great candidate for therapy against *P. aeruginosa.* Further investigation of phage JJ01 in targeting *Pseudomonas* clinical isolates will be needed to explore its potential in therapy.

However, the genome databases of prokaryotic viruses are not fully annotated and many phage-encoding genes in the database remain unknown, rendering it improper to use therapeutically. Rather than using the whole phage, the phage genome is also considered as an untapped resource for antimicrobial discovery (Liu et al., 2004; Van den Bossche et al., 2014; De Smet et al., 2021). Even though phage JJ01 harbors 54 out of 92 genes that were predicted to encode hypothetical proteins, the phage encodes 38 proteins with known functions. We found that the phage encodes putative DNA polymerase III alpha (ORF88) and epsilon subunits (ORF89), suggesting that its DNA metabolism is possibly independent of the host DNA replication system. This also implies that JJ01 might use these subunits to modulate or even interfere with host DNA polymerase during infection which would subsequently impair the host DNA synthesis. Phage JJ01 also harbors five genes that are involved in phage invasion and bacterial cell lysis: *U*-spanin (ORF85) disrupts bacterial outer membrane (Young, 2014), putative endolysin (ORF79) degrades peptidoglycan, gp138 N-terminal-domain containing protein (ORF74) pierces bacterial cell membrane during phage invasion (Browning et al., 2012), transglycosylase SLT domain-containing protein (ORF71) cleaves glycosidic bonds between *N*-acetylglucosamine (NAG) and *N*-acetylmuramic acid (NAM) (Thunnissen et al., 1995), and holin (ORF42) creates holes on the bacterial cell membrane (Young, 2014). Indeed, these antimicrobials potentially support the use of alternative proteins instead of whole phage particles to combat pathogenic bacteria. Further investigation on whether these phage proteins are bactericidal would be needed to explore their antibacterial function.

Since biofilm provides a niche environment for bacteria to embed and serves as a barrier that limits the diffusion of antibiotics to bacterial cells (Drenkard, 2003), the bacteria growing in biofilms are more resistant to antibiotics and the host immune response than the cells that grow freely in suspension (Stewart and William Costerton, 2001). *P. aeruginosa* is one of those that alter its microenvironment by producing biofilms in which, with this protection, it becomes highly resistant to antibiotics up to 1,000 times more than the planktonic cells (Drenkard, 2003; Rumbaugh and Sauer, 2020). In our *in vitro* killing assay, phage JJ01 efficiently suppressed the bacterial growth at various doses even at very low MOI. Even though the emergence of phage resistance can be observed, half of the JJ01-resistant strains isolated in this study clearly exhibit defects in biofilm formation. Some of these strains also lose their ability to grow normally, when compared to the wild type. These characteristics observed in the phage JJ01-resistant strains would be beneficial for therapy. As the bacteria develop resistance to phage and lose the ability to establish biofilms, they would be easier to be eliminated by antibiotics.

A previous study revealed that most phage-resistant bacteria with a reduction of biofilm formation tend to be more susceptible to antibiotics. Moreover, some isolates also had less virulence and were unable to kill *G. mellonella* larvae (Markwitz et al., 2021). This implies that the susceptibility of JJ01-resistant strains to antibiotics might also be altered due to the impaired biofilm-producing ability. According to our antibiotic susceptibility test, W10 and W132, the phage-resistant strains that produce brown pigments (so-called brown phenotype), exhibited eight times higher sensitivity to colistin when compared to the wild type. The brown phenotype as observed in these strains is similar to the previously studied phage-resistant *P. aeruginosa* strains that were isolated during phage PaP1 (Le et al., 2015) and phage PaoP5 (Shen et al., 2018) infections. These mutants had been proven to contain an approximately 200 – 600 kb long genomic fragment deletion that was driven by MutL activity and non-homologous end joining (NHEJ) of the bacteria to avoid phage infection (Shen et al., 2018). The two genes that are always deleted among the brown phenotype mutants include *hmgA*, which is related to the brown pigment accumulation (Rodríguez-Rojas et al., 2009; Hosseinidoust et al., 2013), and *galU*, which is involved in lipopolysaccharide production, bacterial virulence, and synthesis of a component by which the phage recognizes (Le et al., 2015; Pires et al., 2017; Yang et al., 2020). Furthermore, the brown phenotype-producing *P. aeruginosa* strains that are resistant to phages AM.P2, Mat, and Kat have been recently shown to also exhibit hypersusceptibility toward human antimicrobial peptide LL-37 (Menon et al., 2022). Even though the deletion of this particular genomic fragment has been well conserved among the phage-resistant *P. aeruginosa* strains, it is still elusive how the deletion of this genomic region would contribute to the antibiotic hypersensitivity of phage-resistant *P. aeruginosa*. Whole-genome sequencing and analysis of these mutants will be further performed to investigate how the bacteria would trade its physiological fitness, which consequently weakens it, for bacteriophage resistance and results in hypersensitivity to antibiotics.

## Conclusion

Phage JJ01 is a novel lytic bacteriophage that belongs to the *Pbunavirus* genus under the *Myoviridae* family. Phage JJ01 requires about 10 min for 90% adsorption to host cells and 30 min for replication during the latent period, and it produces a relatively large burst size at around 109 particles/cell. It tolerates a wide range of pH and temperature. Although phage-resistant *P. aeruginosa* emerges, it comes at a cost to bacterial survival, by altering some physiological fitness of the bacteria. Some JJ01-resistant isolates produce much less amount of biofilm than the wild type, and some of them become much more susceptible to colistin, which is the last resort antibiotic for Gram-negative bacterial infections. Therefore, phage JJ01 would be considered as a promising candidate for therapeutic purposes in the treatment of *P. aeruginosa* infection.

## Data availability statement

The datasets presented in this study can be found in online repositories. The names of the repository/repositories and accession number(s) can be found in the article/Supplementary material.

## Author contributions

WW, PN, and VC: conceptualization. WW, HH, PN, and VC: methodology. WW, HH, RS, PN, and VC: investigation, formal analysis, and visualization. WW and HH: validation. WW, HH, PN, and VC: writing of the original draft. All authors: reviewing and editing. PN and VC: project administration, resource, and funding acquisition. JP, PN, and VC: supervision.

## Footnotes

1
http://rhea.icbm.uni-oldenburg.de/VIRIDIC/
