# Supplementary material for: Phage-resistant Pseudomonas aeruginosa against a novel lytic phage JJ01 exhibits hypersensitivity to colistin and reduces biofilm production
Source: Front Microbiol. 2022 Oct 6;13:1004733. doi: 10.3389/fmicb.2022.1004733 (PMC9583000; doi:10.3389/fmicb.2022.1004733)
Supplement: Supplementary file 1 [file Data_Sheet_1.PDF]

Supplementary Materials

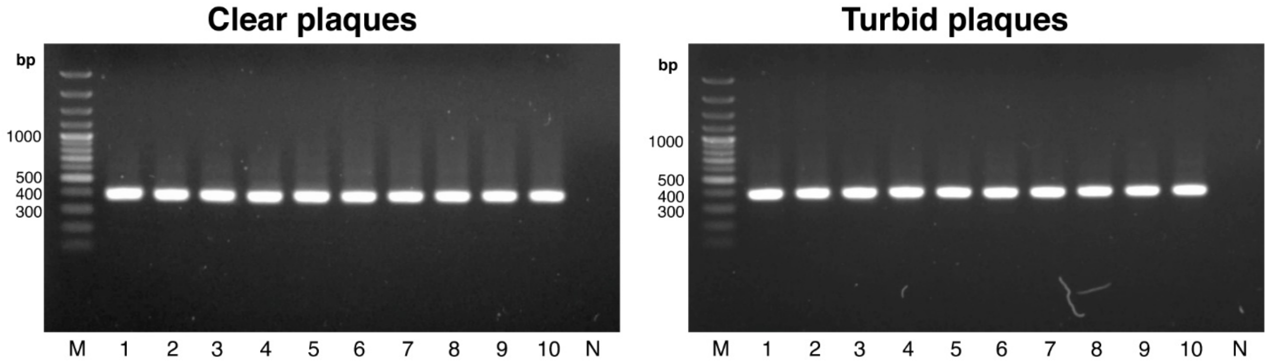

Figure S1. Amplification of gene ORF44 (375 bp) of JJ01 plaques that display different morphologies (clear and turbid plaques). A gel image on the left panel represents ten selected clear plaques while a gel image on the right panel represents ten selected turbid plaques. Numbers beneath the figures refer to plaque number. M is 100 bp DNA marker and N is the negative control.

A

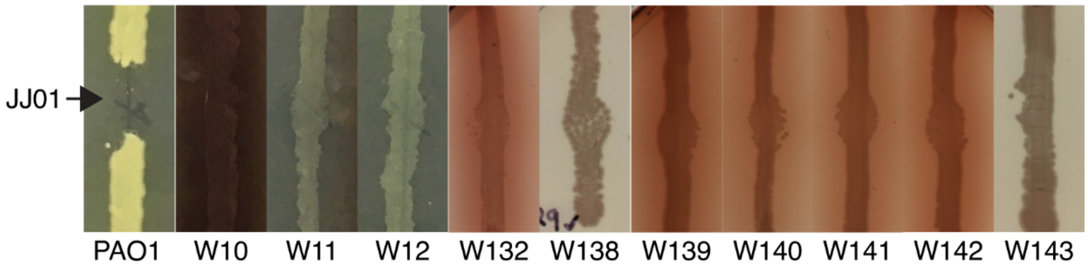

B

Sample name	Instrument/Methods	Results
W10	MALDI Biotyper	*P. aeruginosa*
W11	MALDI Biotyper	*P. aeruginosa*
W12	MALDI Biotyper	*P. aeruginosa*
W132	MALDI Biotyper	*P. aeruginosa*
W138	MALDI Biotyper	*P. aeruginosa*
W139	MALDI Biotyper	*P. aeruginosa*
W140	MALDI Biotyper	*P. aeruginosa*
W141	MALDI Biotyper	*P. aeruginosa*
W142	MALDI Biotyper	*P. aeruginosa*
W143	MALDI Biotyper	*P. aeruginosa*

MALDI Biotype, Bruker Daltonik GmbH, Germany

Figure S2. (A) Stripes of JJ01-resistant *P. aeruginosa* isolates that are resistant to phage JJ01 (black arrow) compared to wildtype PAO1. **(B)** Bacterial identification by MALDI Biotyper confirms that all JJ01-resistant strains are *P. aeruginosa.*

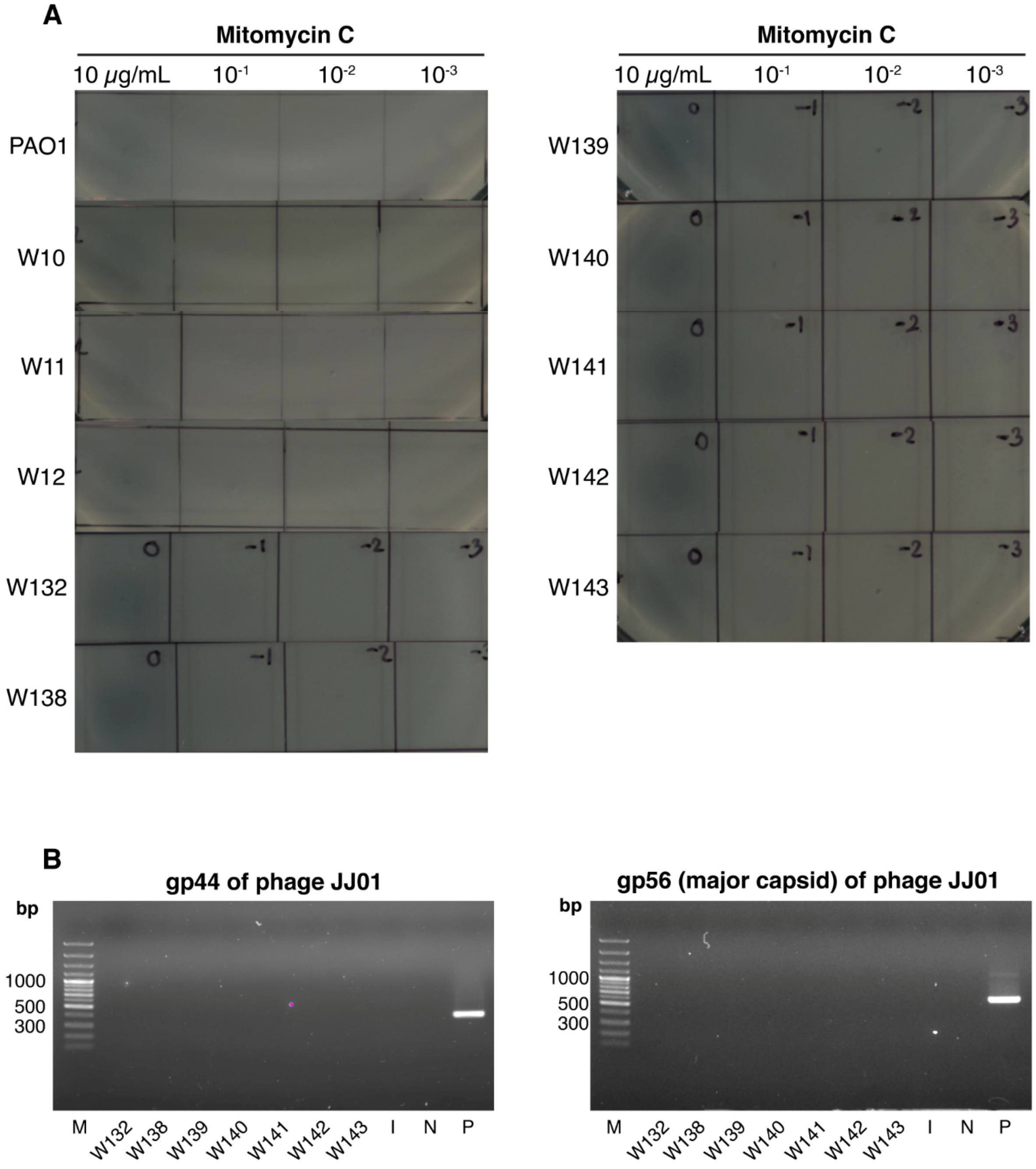

Figure S3. (A) Prophage induction with 10 μg/mL mitomycin C in wildtype PAO1 and JJ01-resistant *P. aeruginosa* isolates. The result revealed that there is no plaque observed in all tested strains. **(B)** Prophage detection in bacterial host genomes by colony PCR of JJ01-resistant isolates with primers specific to JJ01 genes; gp44 (375 bp) and gp56 (544 bp). No positive band appears in all tested JJ01-resistant isolates. M is 100 bp DNA maker. N represents a negative control. I represents an internal control, which PAO1 genome was used as a template, and P is a positive control, which JJ01 genome was used as a template.

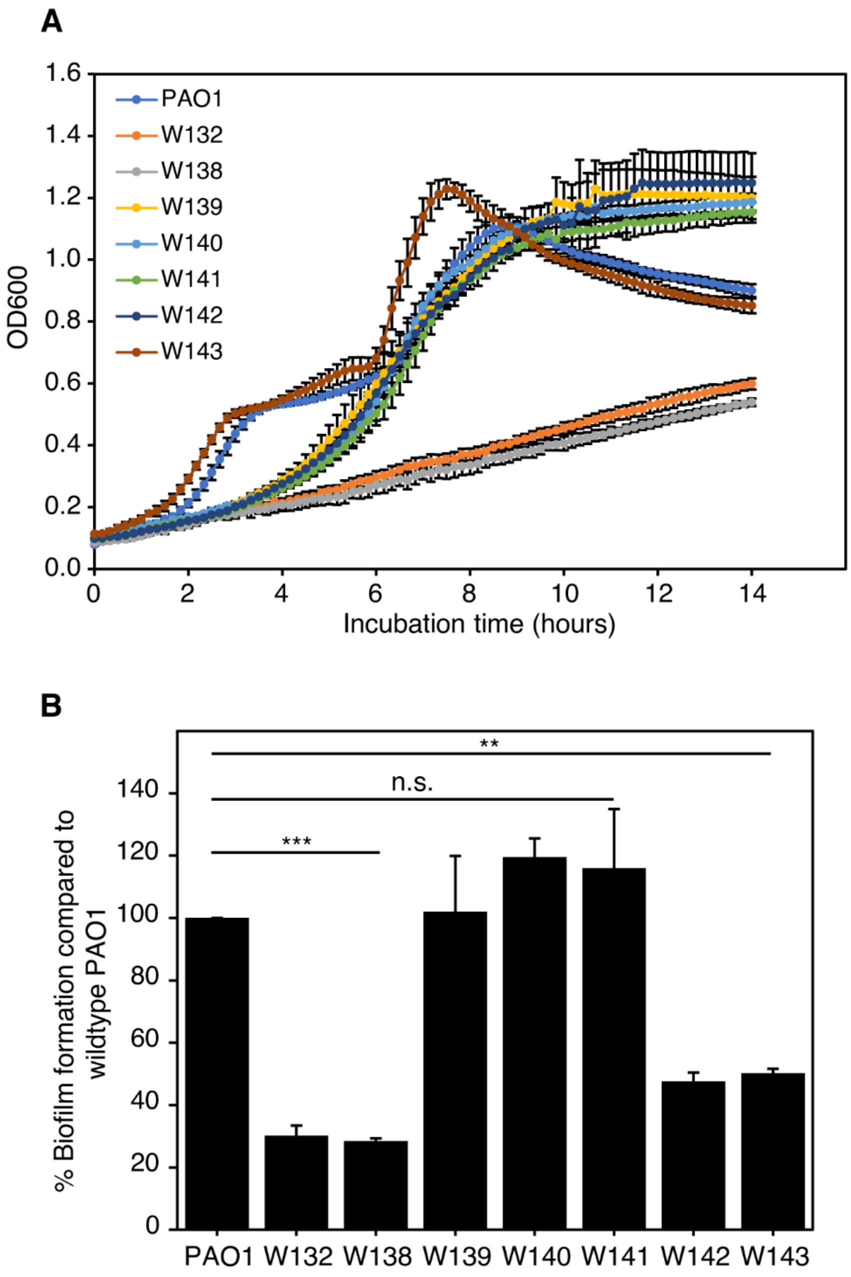

Figure S4. **(A)** Growth curves of 7 additional JJ01-resistant isolates (W132, W138, W139, W140, W141, W142, and W143) compared to wildtype PAO1. **(B)** Biofilm formation assay of JJ01-resistant isolates versus wildtype PAO1. Asterisks (*** and **) represent significant difference at $p \leq 0.001$ and $p \leq 0.01$, respectively. n.s. abbreviated from non-significant difference according to One-way ANOVA followed by Tukey HSD-post hoc tests.

Table S1. VIRIDIC similarity distance (%) and cluster tables between JJ01 and other phages in *Pbunavirus* genus

Genome	KPP12	JJ01	E217	LS1	phiKTN6
KPP12	100	93.89	94.43	95.10	93.63
JJ01	93.89	100	96.04	96.44	95.46
E217	94.43	96.04	100	96.81	94.70
LS1	95.10	96.44	96.81	100	94.68
phiKTN6	93.63	95.46	94.70	94.68	100

Genome	Species cluster	Genus cluster
KPP12	3	1
JJ01	1	1
E217	1	1
LS1	1	1
phiKTN6	2	1

Table S2. List of all annotated ORFs in the genome of phage JJ01. The predicted functions of ORFs were determined by their significant hit (E-value < 10^{-5}) against genome in NCBI and PHASTER.

ORF	Predicted function	Direction	Start	Stop	Size (n)	Sequence similarity	Accession no.	Database
ORF1	Hypothetical protein	-	212	418	207	Hypothetical protein G182_gp59 [*Pseudomonas* phage KPP12]	YP_007238214.1	NCBI
ORF2	Hypothetical protein	-	426	656	231	Hypothetical protein PPSN_gp63 [*Pseudomonas* phage SN]	YP_002418869.1	NCBI
ORF3	Hypothetical protein	-	689	907	219	Hypothetical protein LMA2_gp62 [*Pseudomonas* phage LMA2]	YP_002154296.1	NCBI
ORF4	Hypothetical protein	-	891	1109	219	Hypothetical protein [*Pseudomonas* phage KPP22]	BAU20650.1	NCBI
ORF5	Hypothetical protein	-	1109	1339	231	Hypothetical protein FDG87_gp65 [*Pseudomonas* phage vB_PaeM_CEB_DP1]	YP_009593817.1	NCBI
ORF6	Hypothetical protein	-	1427	2428	1002	Hypothetical protein Epa22_00017 [*Pseudomonas* phage Epa22]	QIQ63984.1	NCBI
ORF7	Head morphogenesis protein	-	2533	3423	891	PHAGE_Pseudo_vB_PaeM_E217_NC_042079: head morphogenesis protein	PP_00007	PHASTER
ORF8	Terminase large subunit	-	3584	4771	1188	PHAGE_Pseudo_vB_PaeM_SCUT_S1_NC_048745: terminase large subunit	PP_00008	PHASTER
ORF9	Hypothetical protein	-	4758	5180	423	Hypothetical protein debbie_69 [*Pseudomonas* phage debbie]	QIQ66568.1	NCBI
ORF10	Terminase small subunit	+	5349	6134	786	PHAGE_Pseudo_vB_PaeM_E217_NC_042079: terminase small subunit	PP_00009	PHASTER
ORF11	Hypothetical protein	+	6145	7374	1230	Hypothetical protein debbie_71 [*Pseudomonas* phage debbie]	QIQ66570.1	NCBI
ORF12	Hypothetical protein	+	7398	7856	459	Hypothetical protein ORF071 [*Pseudomonas* phage F8]	YP_001294488.1	NCBI
ORF13	DNA primase	+	7853	8929	1077	RepB family DNA primase [*Staphylococcus aureus*]	WP_015994935.1	NCBI
ORF14	Hypothetical protein	+	8935	9120	186	Hypothetical protein Pa204_090 [*Pseudomonas* virus Pa204]	QDH46092.1	NCBI
ORF15	DNA helicase	+	9268	11007	1740	DNA helicase [*Pseudomonas* phage Epa20]	QIQ65309.1	NCBI
ORF16	Hypothetical protein	+	11976	12545	570	Hypothetical protein HWB30_gp02 [*Pseudomonas* phage BrSP1]	YP_009835110.1	NCBI
ORF17	Minor tail protein	-	12714	13325	612	PHAGE_Pseudo_vB_PaeM_LS1_NC_048699: minor tail protein	PP_00016	PHASTER

ORF	Predicted function	Direction	Start	Stop	Size (n)	Sequence similarity	Accession no.	Database
ORF18	IgA FC receptor precursor	-	13514	14200	687	IgA FC receptor precursor [*Pseudomonas* phage debbie]	QIQ66578.1	NCBI
ORF19	Hypothetical protein	-	14212	14523	312	Hypothetical protein LBL3 gp76 [*Pseudomonas* phage LBL3]	YP_002154221.1	NCBI
ORF20	Hypothetical protein	-	14576	14806	231	Hypothetical protein HWB54_gp51 [*Pseudomonas* phage vB_PaeM_LS1]	YP_009837399.1	NCBI
ORF21	Hypothetical protein	-	14862	15086	225	Hypothetical protein HWC01_gp84 [*Pseudomonas* phage vB_PaeM_SCUT-S1]	YP_009842984.1	NCBI
ORF22	Hypothetical protein	-	15151	15477	327	Hypothetical protein HWC01 gp85 [*Pseudomonas* phage vB PaeM SCUT-S1]	YP_009842985.1	NCBI
ORF23	Exonuclease	-	15478	16122	645	PHAGE_Pseudo_vB_PaeM_LS1_NC_048699: exonuclease	PP_00022	PHASTER
ORF24	Hypothetical protein	-	16154	16366	213	Hypothetical protein FDJ04_gp88 [*Pseudomonas* phage vB PaeM E217]	YP_009619439.1	NCBI
ORF25	Hypothetical protein	-	16363	16548	186	Hypothetical protein FDJ04_gp89 [*Pseudomonas* phage vB PaeM E217]	YP_009619440.1	NCBI
ORF26	Hypothetical protein	-	16560	16775	216	Hypothetical protein H6S67_gp84 [*Pseudomonas* phage PaGU11]	YP_009913924.1	NCBI
ORF27	Hypothetical protein	-	16772	16972	201	Hypothetical protein H6S67_gp85 [*Pseudomonas* phage PaGU11]	YP_009913925.1	NCBI
ORF28	Hypothetical protein	-	16969	17220	252	Hypothetical protein FDJ04_gp92 [*Pseudomonas* phage vB PaeM E217]	YP_009619443.1	NCBI
ORF29	Hypothetical protein	-	17346	17531	186	Hypothetical protein [*Staphylococcus aureus*]	WP_015992797.1	NCBI
ORF30	DNA adenine methyltransferase	-	17616	17804	189	PHAGE_Pseudo_vB_PaeM_LS1_NC_048699: DNA adenine methyltransferase	PP_00030	PHASTER
ORF31	Tail length tape-measure protein	-	17807	18493	687	Tail length tape-measure protein [*Pseudomonas* phage debbie]	QIQ66591.1	NCBI
ORF32	Methyltransferase type 11	-	18546	18848	303	PHAGE_Pseudo_vB_PaeM_SCUT_S1_NC_048745 : methyltransferase type 11	PP_00032	PHASTER
ORF33	Hypothetical protein	-	18859	19005	147	Hypothetical protein [*Pseudomonas* phage Epa15]	QJQ38355.2	NCBI
ORF34	Putative phosphoesterase	-	19203	19367	165	PHAGE_Pseudo_vB_PaeM_LS1_NC_048699: putative phosphoesterase	PP_00035	PHASTER
ORF35	Phage protein	-	19410	19604	195	Phage protein [*Pseudomonas* phage S50]	BBJ26915.1	NCBI

ORF	Predicted function	Direction	Start	Stop	Size (n)	Sequence similarity	Accession no.	Database
ORF36	Putative terminase	+	19772	21154	1383	Putative terminase [*Pseudomonas* phage KPP12]	YP_007238156.1	NCBI
ORF37	Hypothetical protein	-	21191	21556	366	Hypothetical protein [*Pseudomonas* phage Epa15]	QJQ38320.2	NCBI
ORF38	Hypothetical protein	-	21574	21789	216	Hypothetical protein HWB30_gp22 [*Pseudomonas* phage BrSP1]	YP_009835130.1	NCBI
ORF39	Hypothetical protein	-	21789	22139	351	Hypothetical protein [*Pseudomonas* phage PHW2]	QKW95252.1	NCBI
ORF40	Hypothetical protein	-	22183	22566	384	Hypothetical protein Pa204_026 [*Pseudomonas* virus Pa204]	QDH46028.1	NCBI
ORF41	DUF2786 protein	-	22569	23348	780	[pfam10979] cl12553 (PSSM Id: 402523) Protein of unknown function	cl12553	NCBI Conserved Domain Search
ORF42	Holin	-	23435	23872	438	PHAGE_Pseudo_Epa13_NC_050147: holin	PP_00043	PHASTER
ORF43	Hypothetical protein	-	23889	24476	588	MULTISPECIES: Hypothetical protein [Bacteria]	WP_015992645.1	NCBI
ORF44	Hypothetical protein	-	24578	25510	933	Hypothetical protein debbie_12 [*Pseudomonas* phage debbie]	QIQ66511.1	NCBI
ORF45	Hypothetical protein	-	25614	25961	348	Hypothetical protein FDJ04_gp14 [*Pseudomonas* phage vB_PaeM_E217]	YP_009619365.1	NCBI
ORF46	Hypothetical protein	-	26210	26527	318	Hypothetical protein [*Pseudomonas* phage Epa15]	QPL17318.1	NCBI
ORF47	Hypothetical protein	-	26527	26730	204	Hypothetical protein HWB30_gp32 [*Pseudomonas* phage BrSP1]	YP_009835140.1	NCBI
ORF48	Hypothetical protein	-	26727	27050	324	Hypothetical protein AVT16_gp19 [*Pseudomonas* phage vB_Pae_PS44]	YP_009211343.1	NCBI
ORF49	Phage protein	-	27082	27483	402	Phage protein [*Pseudomonas* phage PA01]	YP_009829582.1	NCBI
ORF50	Minor capsid protein	+	27664	29961	2298	Minor capsid protein [*Pseudomonas* phage vB_PaeM_USP_1]	YP_009914222.1	NCBI
ORF51	Phage Mu protein F like protein	+	29961	30797	837	[Phage_Mu_F super family] cl10072 (PSSM Id: 415838) Phage Mu protein F like protein	cl10072	NCBI Conserved Domain Search
ORF52	Hypothetical protein	+	30816	31022	207	Hypothetical protein FDG87_gp21 [*Pseudomonas* phage vB_PaeM_CEB_DP1]	YP_009593773.1	NCBI
ORF53	Hypothetical protein	+	31019	31159	141	Hypothetical protein [*Staphylococcus aureus*]	WP_174840982.1	NCBI
ORF54	DUF2213 protein	+	31672	33105	1434	[DUF2213 super family] cl19842 (PSSM Id: 418671) Uncharacterized protein conserved in bacteria	cl19842	NCBI Conserved Domain Search

ORF	Predicted function	Direction	Start	Stop	Size (n)	Sequence similarity	Accession no.	Database
ORF55	Hypothetical protein	+	33109	33744	636	Hypothetical protein HWB54_gp16 [*Pseudomonas* phage vB_PaeM_LS1]	YP_009837364.1	NCBI
ORF56	DUF2184 protein (major capsid)	+	33754	34902	1149	[DUF2184 super family] cl21556 (PSSM Id: 419730) Uncharacterized protein conserved in bacteria	cl21556	NCBI Conserved Domain Search
ORF57	Hypothetical protein	+	35004	35441	438	Hypothetical protein [*Staphylococcus aureus*]	WP_015992658.1	NCBI
ORF58	DUF4054 domain-containing protein	+	35456	35923	468	DUF4054 domain-containing protein [*Pseudomonas aeruginosa*]	WP_016066139.1	NCBI
ORF59	Putative structural protein	+	35920	36318	399	Putative structural protein [*Staphylococcus aureus*]	WP_016066140.1	NCBI
ORF60	Structural protein	+	36326	36877	552	Structural protein [*Pseudomonas* phage phiKTN6]	YP_009593265.1	NCBI
ORF61	Hypothetical protein	+	36874	37455	582	Hypothetical protein ORF028 [*Pseudomonas* phage F8]	YP_001294445.1	NCBI
ORF62	DUF3383 domain-containing protein	+	37471	38985	1515	DUF3383 domain-containing protein [*Staphylococcus aureus*]	WP_174840936.1	NCBI
ORF63	Hypothetical protein	+	39044	39496	453	MULTISPECIES: Hypothetical protein [Bacteria]	WP_015992745.1	NCBI
ORF64	Hypothetical protein	+	39496	39819	324	MULTISPECIES: Hypothetical protein [Bacteria]	WP_015992746.1	NCBI
ORF65	Hypothetical protein	+	39816	40166	351	MULTISPECIES: Hypothetical protein [Bacteria]	WP_015992747.1	NCBI
ORF66	Hypothetical protein	+	40168	40599	432	Hypothetical protein 25_00005 [*Pseudomonas* phage Epa25]	QIQ65544.1	NCBI
ORF67	Putative structural protein	+	40609	41112	504	Putative structural protein [*Pseudomonas* phage KPP12]	YP_007238188.1	NCBI
ORF68	Structural protein	+	41247	41651	405	Structural protein [*Pseudomonas* phage goonie]	QJB23037.1	NCBI
ORF69	Hypothetical protein	+	41660	42253	594	Hypothetical protein [*Staphylococcus aureus*]	WP_016066145.1	NCBI
ORF70	Hypothetical protein	+	42263	42691	429	Hypothetical protein [*Staphylococcus aureus*]	WP_174840938.1	NCBI
ORF71	Transglycosylase SLT domain-containing protein	+	42695	45271	2577	Transglycosylase SLT domain cl00222 (PSSM Id: 396169)	cl00222	NCBI Conserved Domain Search
ORF72	Hypothetical protein	+	45271	46134	864	Hypothetical protein [*Staphylococcus aureus*]	WP_016066147.1	NCBI
ORF73	Hypothetical protein	+	46134	46667	534	Hypothetical protein [*Staphylococcus aureus*]	WP_015994906.1	NCBI
ORF74	Gp138 N-terminal domain-containing protein	+	46723	47388	666	[Gp138_N super family] cl39697 (PSSM Id: 423479) Phage protein Gp138 N-terminal domain	cl39697	NCBI Conserved Domain Search

ORF	Predicted function	Direction	Start	Stop	Size (n)	Sequence similarity	Accession no.	Database
ORF75	Baseplate J/gp47 family protein	+	47446	48699	1254	Baseplate J/gp47 family protein [*Pseudomonas aeruginosa*]	MBI7739469.1	NCBI
ORF76	DUF2612 protein	+	48696	50210	1515	[DUF2612 super family] cl12607 (PSSM Id: 416601) Protein of unknown function	cl12607	NCBI Conserved Domain Search
ORF77	Hypothetical protein	+	50215	53109	2895	Hypothetical protein FDJ04_gp46 [*Pseudomonas* phage vB_PaeM_E217]	YP_009619397.1	NCBI
ORF78	Hypothetical protein	+	53111	53539	429	Hypothetical protein H6S68_gp90 [*Pseudomonas* phage Epa7]	YP_009914020.1	NCBI
ORF79	Putative endolysin	+	53539	54201	663	Putative endolysin [*Pseudomonas* phage KPP12]	YP_007238200.1	NCBI
ORF80	Hypothetical protein	-	54226	54477	252	Hypothetical protein [*Staphylococcus aureus*]	WP_015994912.1	NCBI
ORF81	DNA ligase	-	54757	55668	912	DNA ligase [*Pseudomonas* phage PHW2]	QKW95294.1	NCBI
ORF82	DNA-binding protein	-	55723	56277	555	DNA-binding protein [*Pseudomonas* phage debbie]	QIQ66549.1	NCBI
ORF83	Hypothetical protein	-	56274	56879	606	Hypothetical protein PP141_gp51 [*Pseudomonas* phage 14-1]	YP_002364359.1	NCBI
ORF84	Hypothetical protein	-	56936	57835	900	Hypothetical protein jett_51 [*Pseudomonas* phage jett]	QIQ66830.1	NCBI
ORF85	U-spanin	-	57924	58544	621	PHAGE_Pseudo_vB_PaeM_E215_NC_042080: u-spanin	PP_00086	PHASTER
ORF86	SNF2 family N-terminal domain-containing protein	-	58639	60198	1560	[SNF2_N Superfamily] cl37620 (PSSM Id: 422040) SNF2 family N-terminal domain	cl37620	NCBI Conserved Domain Search
ORF87	Putative DNA helicase	-	60195	60605	411	Putative DNA helicase [*Pseudomonas* phage NH-4]	YP_007002602.1	NCBI
ORF88	DNA polymerase III subunit alpha	-	60598	63708	3111	DNA polymerase III subunit alpha [*Staphylococcus aureus*]	WP_174840946.1	NCBI
ORF89	Putative DNA polymerase III epsilon subunit	-	63705	64259	555	Putative DNA polymerase III epsilon subunit [*Pseudomonas* phage NH-4]	YP_007002604.1	NCBI
ORF90	Hypothetical protein	-	64335	65354	1020	Hypothetical protein H6S67_gp57 [*Pseudomonas* phage PaGU11]	YP_009913897.1	NCBI
ORF91	Hypothetical protein	-	65357	65548	192	Hypothetical protein [*Pseudomonas* phage Epa15]	QPL17331.1l	NCBI
ORF92	Putative thymidylate synthase	-	65550	66338	789	Putative thymidylate synthase [*Pseudomonas* phage PaGU11]	YP_009913899.1	NCBI
